# Characterization of diffusion magnetic resonance imaging revealing relationships between white matter disconnection and behavioral disturbances in mild cognitive impairment: a systematic review

**DOI:** 10.3389/fnins.2023.1209378

**Published:** 2023-06-08

**Authors:** Yu Zhou, Lan Wei, Song Gao, Jun Wang, Zhigang Hu

**Affiliations:** ^1^College of Medical Technology and Engineering, Henan University of Science and Technology, Luoyang, China; ^2^Business School, The University of Sydney, Sydney, NSW, Australia; ^3^College of Agricultural Equipment Engineering, Henan University of Science and Technology, Luoyang, China; ^4^School of Information Engineering, Henan University of Science and Technology, Luoyang, China

**Keywords:** mild cognitive impairment, diffusion MRI, white matter disconnection, cognition, affection

## Abstract

White matter disconnection is the primary cause of cognition and affection abnormality in mild cognitive impairment (MCI). Adequate understanding of behavioral disturbances, such as cognition and affection abnormality in MCI, can help to intervene and slow down the progression of Alzheimer’s disease (AD) promptly. Diffusion MRI is a non-invasive and effective technique for studying white matter microstructure. This review searched the relevant papers published from 2010 to 2022. Sixty-nine studies using diffusion MRI for white matter disconnections associated with behavioral disturbances in MCI were screened. Fibers connected to the hippocampus and temporal lobe were associated with cognition decline in MCI. Fibers connected to the thalamus were associated with both cognition and affection abnormality. This review summarized the correspondence between white matter disconnections and behavioral disturbances such as cognition and affection, which provides a theoretical basis for the future diagnosis and treatment of AD.

## Introduction

###  The significance of research for behavioral disturbances in MCI

**FIGURE 1:**
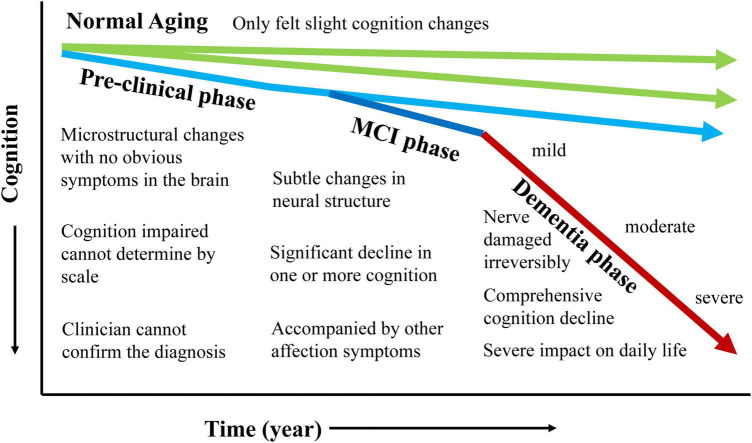
The progression of Alzheimer’s disease for each phase.

Mild cognitive impairment (MCI) is the prodromal stage of Alzheimer’s disease (AD) (Petersen et al., 1999; Gauthier et al., 2006). As the progression of AD showed in Figure 1, the neuronal destruction in the AD stage is so extensive for the whole brain that it is difficult to reverse. Many treatments are only effective for MCI with subtle changes in neural structure.

Moving the development window for biomarkers forward to the MCI stage can halt or slow AD progression (Wang et al., 2013). Therefore, the MCI stage is the best opportunity for intervention in AD timely.

Mild cognitive impairment often presents with behavioral disturbances, mainly memory loss, reduced attention and executive functions, disorientation, and impaired language skills, collectively referring to cognition decline (Catani et al., 2013; Arvanitakis et al., 2019). In addition, MCI often have other affective symptoms, including depression, anxiety, and apathy (Catani et al., 2012). These affective symptoms may exacerbate the transition from MCI to AD. Therefore, fully understanding the behavioral disturbances in MCI can help promptly intervene and delay AD development (Pantel et al., 2016).

### White matter disconnections caused behavioral disturbances in MCI

The behavioral disturbances in MCI are mainly caused by the disconnection of neuronal pathways in the brain due to white matter degeneration (Alves et al., 2017; Yu et al., 2021). During the development of AD, the lesioned areas propagate from the lower to the higher cortex according to specific white matter pathways (Gainotti et al., 2014; Miller et al., 2016). During the MCI stage, selective degeneration in fibers is mainly in the limbic system (Mito et al., 2018). In the late AD stage, fibers gradually spread from the limbic system to the higher cortices, such as frontal, temporal and parietal, for extensive degeneration throughout the brain (Pini et al., 2016; Zimmermann et al., 2018).

White matter connections between the hippocampus, precuneus and posterior cingulate cortex form the memory network. The Papez circuit formed between the hippocampus and thalamus has also been shown to be related to working memory (Li K. et al., 2020). The arcuate fasciculus, which connects the frontal Broca’s area to the temporal Veronica area, is associated with language ability (Friederici and Gierhan, 2013). The inferior longitudinal fasciculus and the inferior frontal-occipital fasciculus, which runs through the temporal lobe and reaches the occipital lobe, are associated with visuospatial ability (Urbanski et al., 2008). Damage to the white matter of these fibers in MCI leads to cognitive impairment.

Besides memory deficits, MCI has executive function deficits such as attention and information processing speed (Saunders and Summers, 2011). As a relay station for transmitting information from subordinate neurons to the cerebral cortex, the thalamus has extensive white matter fiber connections from the subcortical nuclei to the cerebral cortex (Abivardi and Bach, 2017; Zheng et al., 2019). In addition to the Papez circuit with the hippocampus and other subcortical nuclei in the limbic systems, the thalamus is responsible for memory processing (Bubb et al., 2017). The thalamus is also connected to the frontal and parietal cortex via projection fibers (Gerstenecker et al., 2017), which regulate cognition and affection (Gu and Zhang, 2019).

In addition, there are overlaps and interactions on some neural pathways between brain networks related to cognition and affection in MCI (Tan et al., 2019), which may be essential in converting MCI to AD (Barca et al., 2017; Sui et al., 2020). However, current research has focused on the relationship between white matter damage and cognition decline in MCI. But it is unclear which fibers are associated with affection symptoms in MCI. The comorbid pathways of brain networks related to cognition and affection in MCI are indistinct. Therefore, it is necessary for relevant studies to organize and summarize the relationship between white matter disconnections and behavioral disturbances in MCI.

### Parameters of diffusion MRI could explain white matter disconnections effetely

**FIGURE 2:**
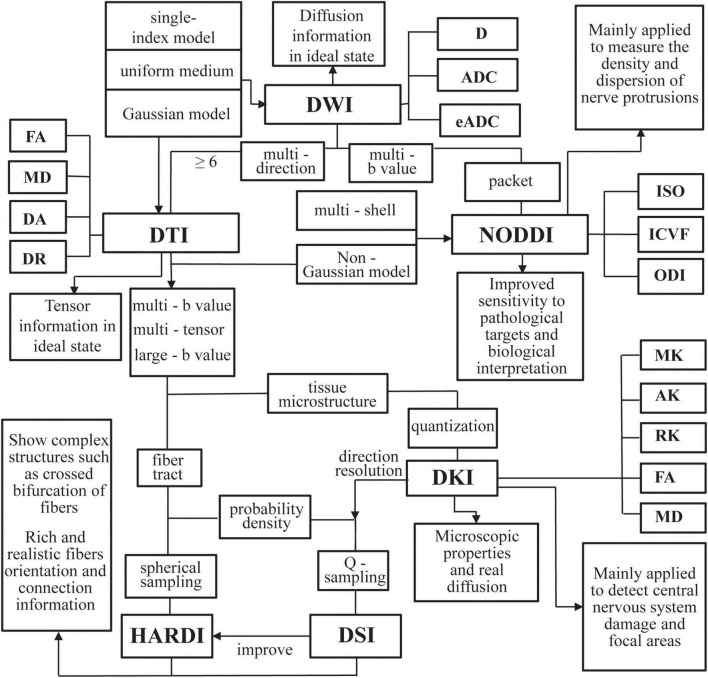
An overview of the advanced diffusion magnetic resonance imaging (MRI) techniques examined in this article. DWI, diffusion weighted imaging; D, diffusion; ADC, apparent diffusion coefficient; eADC, exponential ADC; DTI, diffusion tensor imaging; FA, fractional anisotropy; DA, axial diffusivity; DR, radial diffusivity; MD, mean diffusivity; DSI, diffusion spectrum imaging; HARDI, high angular diffusion magnetic imaging; DKI, diffusion kurtosis imaging; MK, mean kurtosis; AK, axial kurtosis; RK, radial kurtosis; NODDI, neurite orientation dispersion and density imaging; ODI, orientation dispersion index; ICVF, intracellular volume fraction; ISO, isotropic volume fraction.

Due to the sensitivity of the diffusion MRI signals for the moving of water molecules, it can effectively probe tissue microstructures. The water molecules’ movement is restricted and obstructed by the fiber structure of neuronal axons. So the motion trail of the water molecules can be used to infer intra-voxel fiber orientation and outline the path of white matter by using appropriate fiber tracking algorithms. Diffusion tensor imaging (DTI) focuses on obtaining reliable indicators of key microstructural parameters. The fractional anisotropy (FA) is quantitatively described using the proportion of diffusion anisotropy included in the diffusion tensor, reflecting the integrity of the protein fibers’ myelin sheath and density (Richards et al., 1992). The axial diffusivity (DA) represents the diffusion rate of water molecules along the central axis and is usually used to reflect the degeneration of axons (Alexander et al., 2007). Radial diffusivity (DR) could reflect the permeability of water molecules along the radial direction (Song et al., 2002). Mean diffusivity (MD) indicates the average diffusivity of water molecules in brain tissue (Le et al., 2001). Based on DTI, diffusion kurtosis imaging (DKI), diffusion spectrum imaging (DSI) and neurite orientation dispersion and density imaging (NODDI) have been developed (Pasternak et al., 2018). The advanced diffusion MRI techniques were showed in Figure 2. The parameters of diffusion imaging can be used to quantitatively characterize the degeneration of white matter and further analyze the relationship between the white matter disconnection and behavioral disturbances such as cognitive and emotional disorders in MCI.

### Contribution of this article

Despite the outstanding achievements of neuroimaging methods for white matter, it remains unclear which fibers’ degeneration causes cognition and affection abnormalities, and whether these white matter disconnections are associated with different patterns and severity of behavioral disturbances. For this purpose, our review combined with evidence of white matter disconnections and behavioral disturbances in MCI, provided more insight by integrating and analyzing all studies of white matter from diffusion MRI methods.

The current work aims to review diffusion MRI findings of behavioral disturbances in MCI, focusing on the relationship between diffusion parameters of white matter and behavioral scores. We acknowledge that the number of studies conducted to date is not significant. For the second purpose, our review critically discussed the comorbid pathways related to cognition and affection according to the neurobiological mechanisms in MCI.

In summary, this review provided new development in how diffusion imaging methods have been used for cognition and affection symptoms in MCI. The potential role of neuroimaging evidence was highlighted for the early diagnosis of AD. The basis could be provided for the targeted treatment of specific fiber tracts.

## Methods

The present research review followed the Problem Intervention Comparison Outcomes (PICO) search strategy based on the brain mechanisms of white matter disconnections related to behavioral disturbances in MCI. Diffusion imaging characteristics of white matter in MCI were summarized and compared with healthy controls. The relationship between different brain regions’ white matter disconnections and behavioral disturbances was discussed.

In this review, PubMed and Web of Science databases were systematically searched for relevant literature from 2010 to 2022. Three sets of keywords were used for the literature search: (i) mild cognitive impairment; (ii) white matter; (iii) behavior. Keywords included in the title or abstract of the paper are also included in this review. In addition to the systematic electronic database search, a targeted search of the bibliographies of relevant articles was conducted to identify any additional papers to be included.

Only original articles published in English between January 2010 and December 2022 were considered. All articles investigated the relationship between white matter disconnections and behavioral disturbances in MCI through diffusion MRI methods. Articles were excluded if they: (i) did not use diffusion MRI to investigate the white matter; (ii) studied other disorders such as cerebrovascular disease, sclerosis, hypertension, cerebral infarction, stroke, Parkinson’s, Lewy body dementia, Down syndrome, and schizophrenia; (iii) were review articles.

## Results

### Search results

**FIGURE 3:**
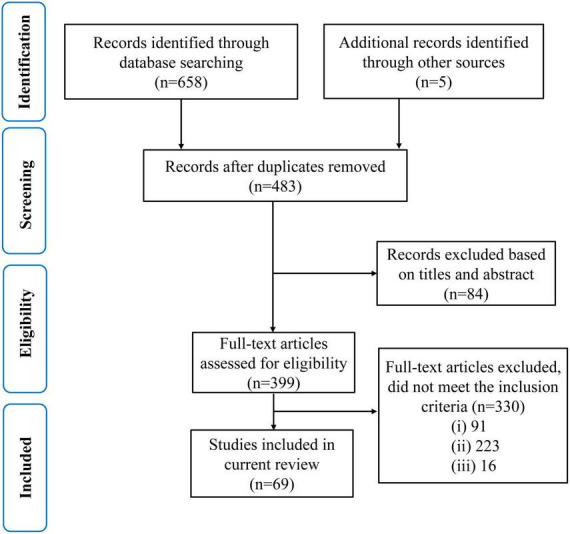
A modified preferred reporting items for systematic reviews and meta-analyses (PRISMA) flow diagram through the selection study.

Using the search method mentioned above, a total of 479 articles were retrieved from the Web of Science database, 179 from the PubMed database, and five from other databases. After the initial screening, duplicate and irrelevant papers were removed. 330 articles were excluded according to the exclusion criteria. 91 articles investigating the white matter without diffusion MRI were excluded based on case (i); 223 articles (55 in cerebrovascular disease, three in sclerosis, three in hypertension, nine in cerebral infarction, 69 in stroke, 70 in Parkinson’s, 10 in Lewy body dementia, three in Down syndrome, one in schizophrenia) were excluded based on case (ii), and 16 review articles were excluded based on case (iii). Finally, 69 articles were selected for this review. The preferred reporting items for systematic reviews and meta-analyses (PRISMA) diagram in Figure 3 illustrates the screening and inclusion process.

### Behavior testing scales

The different behaviors of the subjects were divided into cognition testing scales as well as affection testing scales. The scales used to test various cognition for MCI are summarized in Table 1, including memory, language, visual space, attention and execution. Table 2 summarizes the scales used to test different affections in MCI, including depression, anxiety, and apathy.

**TABLE 1:** Cognition testing scales.

Cognition	Testing scale	Literature
Memory	Hopkins verbal learning test (HVLT)	Shi et al., 2012
	California verbal learning test (CVLT)	Rabin et al., 2009
	Rey auditory verbal learning test (RAVLT)	Gainotti et al., 1998
	Delayed story recall (DSR)	Shi et al., 2014
	Free and cued selective recall test (FCSRT)	Sarazin, 2008
	Categorical cue recall (CCR)	Vogel et al., 2007
Attention and execution	Trail making test-A/B (TMT-A/B)	Ashendorf et al., 2008
	Victoria stroop test (VST)	Bayard et al., 2011
	Wisconsin card sorting Test (WCST)	Paolo et al., 1996
Visual space	Clock drawing test (CDT)	Lee et al., 1996
	Rey-Osterrieth complex figure test (ROCFC)	Reedy et al., 2013
Language	Verbal fluency test (VFT)	Torralva et al., 2015
	Boston naming test (BNT-12)	Serrano et al., 2001
	Grading naming test (GNT)	Ahmed et al., 2008
	Controlled Verbal Word Association Test (COWAT)	Kohnert et al., 1998

**TABLE 2:** Affection testing scales.

Affection	Testing scale	Literature
Depression	Geriatric Depression Scale (GDS)	Burke et al., 1991
Anxiety	Neuropsychiatric Inventory Questionnaire (NPI-Q)	Boada et al., 2002
	Hamilton Anxiety Scale (HAMA)	Thompson, 2015
Indifference	Neuropsychiatric Inventory Questionnaire (NPI-Q)	Boada et al., 2002
	Dimensional Apathy Scale (DAS)	Radakovic and Abrahams, 2014

### Assessment of white matter disconnection and behavioral disturbances

The fibers were divided into four groups according to their location and function. The four groups were limbic, projection, association, and commissural fibers (Table 3). Research on the correlation between cognition testing scale scores and diffusion parameters of limbic, projection, association, and commissural fibers in MCI were summarized in Tables 4–7, respectively. Research on the correlation between affection testing scale scores and diffusion parameters of limbic and projection fibers were summarized in Tables 8, 9. The following patterns can be found in the tables. Firstly, the limbic, association and commissural fibers were mainly related to cognition. Secondly, the limbic and projection fibers were primarily related to affection. Finally, the limbic fibers were associated with both cognition and affection.

**TABLE 3:** Classification of fibers.

Attribution	Full name of fiber	Abbreviation
Limbic fibers	Anterior cingulum	aCingulum
	Posterior cingulum	pCingulum
	Fornix	Fornix
	Uncinate fasciculus	UF
	Anterior thalamic radiation	ATR
Projection fibers	Anterior corona radiata	ACR
	Superior corona radiata	SCR
	Posterior corona radiata	PCR
	Posterior limbs of internal capsules	PLIC
	Retrolenticular part of internal capsule	RIC
	Cerebellar peduncle	CP
Association fibers	Superior longitudinal fasciculus	SLF
	Inferior fronto-occipital fasciculus	IFOF
	Inferior longitudinal fasciculus	ILF
Commissural fibers	Genu of corpus callosum	GCC
	Body of corpus callosum	BCC
	Splenium of corpus callosum	SCC

**TABLE 4:** Correlation analysis between white matter parameters and cognition scales in limbic fibers.

Cognition			Fibers		
	**aCingulum**	**pCingulum**	**Fornix**	**UF**	**ATR**
Episodic memory	–	*DTI (FA*↓*;MD,DA,DR↑)* (Li K. et al., 2020) (Li X. et al., 2020) (Jung et al., 2018) (Gyebnar et al., 2018) (Chang et al., 2016) (Ray et al., 2015) (Remy et al., 2015) (Nir et al., 2013) (Metzler-Baddeley et al., 2012a) (Zhuang et al., 2012a) (Bozzali et al., 2012) (Sexton et al., 2010) (Jhoo et al., 2010) *DTI* + *DSI* (Lin et al., 2014) *DTI* + *networks* (Berlot et al., 2016) (Carter et al., 2014) *DTI* + *NAA* (Wong et al., 2020) *Volume* (Li et al., 2016)	*DTI (FA*↓*;MD,DA,DR↑)* (Zhou et al., 2022a) (Yu et al., 2020) (Ray et al., 2015) (Remy et al., 2015) (Boespflug et al., 2014a) (Boespflug et al., 2014b) (Metzler-Baddeley et al., 2012a) (Lee et al., 2012) (Mielke et al., 2012) (Sexton et al., 2010) *DTI* + *tract length* (Srisaikaew et al., 2020) *Volume* (Li et al., 2016)	*DTI (FA*↓*;MD,DA,DR↑)* (Fan et al., 2018) (Remy et al., 2015) (Carter et al., 2014) (Metzler-Baddeley et al., 2012a) (Serra et al., 2012) (O’Dwyer et al., 2011) *DTI* + *number* (Park et al., 2019) *Volume* (Li et al., 2016)	*DTI (FA*↓*;MD,DA,DR↑)* (Zhou et al., 2022a) (Chen et al., 2020) *Volume* (Benavides-Varela et al., 2020)
Semantic memory	*DTI (FA*↓*;MD,DA,DR↑)* (Dimitra et al., 2013) (Metzler-Baddeley et al., 2012b) (Delano-Wood et al., 2012)	*DTI (FA*↓*;MD,DA,DR↑)* (Gyebnar et al., 2018) (Dimitra et al., 2013) *DTI* + *NODDI* (Wen et al., 2019) *DTI* + *network* (Kim et al., 2019) *DTI* + *tract number* (Park et al., 2019)	*DTI (FA*↓*;MD,DA,DR↑)* (Gyebnar et al., 2018) (Zhuang et al., 2012b) *DTI* + *A*β (Egli et al., 2015)	*DTI* + *network* (Healey et al., 2021) *DTI* + *tract number* (Park et al., 2019)	*Volume* (Benavides-Varela et al., 2020)
Visuospatial	*DTI (FA*↓*;MD,DA,DR↑)* (Dimitra et al., 2013) (Metzler-Baddeley et al., 2012b)	*DTI (FA*↓*;MD,DA,DR↑)* (Gyebnar et al., 2018) (Dimitra et al., 2013) *DTI* + *DSI* (Lin et al., 2014) *DTI* + *tract number* (Park et al., 2019)	*DTI (FA*↓*;MD,DA,DR↑)* (Gyebnar et al., 2018) (Christiansen et al., 2016)	*DTI* + *tract number* (Park et al., 2019)	*Volume* (Benavides-Varela et al., 2020)
Attention and executive functions	–	*DTI (FA*↓*;MD,DA,DR↑)* (Gill et al., 2021) (Li X. et al., 2020) (Ray et al., 2015) (Metzler-Baddeley et al., 2012b) *DTI* + *DSI* (Lin et al., 2014) *DTI* + *networks* (Berlot et al., 2016)	*DTI* + *tract length* (Srisaikaew et al., 2020)	*DTI (FA*↓*;MD,DA,DR↑)* (Gill et al., 2021) (Serra et al., 2012)	–

**TABLE 5:** Correlation analysis between white matter parameters and cognition scales in projection fibers.

Cognition				Fibers		
**CP**	**ACR**	**SCR**	**PCR**	**PLIC**	**RIC**	**CP**
Episodic memory	*Volume* (Fujishima et al., 2014)	–	*Volume* (Fujishima et al., 2014)	*DTI (FA↑;MD,DA,DR↑)* (Zimny et al., 2012)	*DTI (FA↑;MD,DA,DR↑)* (Shim et al., 2017)	*DTI (FA↑;MD,DA,DR↑)* (Mascalchi et al., 2019)

**TABLE 6:** Correlation analysis between white matter parameters and cognition scales in association fibers.

Cognition		Fibers	
	**SLF**	**IFOF**	**ILF**
Episodic memory	*DTI (FA*↓*;MD,DA,DR↑)* (Chen et al., 2020) (Hsu et al., 2019) (Yang et al., 2021) (Qin et al., 2016) (Carter et al., 2014) (Douaud et al., 2013) *DTI* + *NODDI* (Fu et al., 2020)	*DTI (FA*↓*;MD,DA,DR↑)* (Chen et al., 2020) (Carter et al., 2014) (Bosch et al., 2012) *DTI* + *volume* (Gao et al., 2019)	*DTI (FA*↓*;MD,DA,DR↑)* (Zhou et al., 2022a) (Chen et al., 2020) (Bosch et al., 2012) *DTI* + *volume* (Gao et al., 2019)
Semantic memory	*DTI (FA*↓*;MD,DA,DR↑)* (Dimitra et al., 2013) *DTI* + *network* (Healey et al., 2021)	–	–
Visuospatial	*DTI (FA*↓*;MD,DA,DR↑)* (Dimitra et al., 2013)	–	–
Attention and executive functions	*DTI* + *network* (Farrar et al., 2018)	*DTI (FA*↓*;MD,DA,DR↑)* (Snir et al., 2019) *DTI* + *network* (Farrar et al., 2018) *DTI* + *volume* (Gao et al., 2019)	*DTI (FA*↓*;MD,DA,DR↑)* (Farrar et al., 2018) *DTI* + *volume* (Gao et al., 2019)

**TABLE 7:** Correlation analysis between white matter parameters and cognition scales in commissural fibers.

Cognition		Fibers	
	**GCC**	**BCC**	**SCC**
Episodic memory	*DTI (FA*↓*;MD,DA,DR↑)* (Raghavan et al., 2020) (Hsu et al., 2019) (Jiang et al., 2018) *DTI* + *DKI* (Allen et al., 2019) *DTI* + *network* (Li et al., 2016)	*DTI (FA*↓*;MD,DA,DR↑)* (Li et al., 2016)	*DTI (FA*↓*;MD,DA,DR↑)* (Jiang et al., 2018) (Zhang et al., 2011) *DTI* + *network* (Rieckmann et al., 2016)
Semantic memory	*DTI (FA*↓*;MD,DA,DR↑)* (Dimitra et al., 2013) (Grambaite et al., 2010)	*Volume* (Ansado et al., 2013)	*DTI (FA*↓*;MD,DA,DR↑)* (Delano-Wood et al., 2010)
Visuospatial	*DTI (FA*↓*;MD,DA,DR↑)* (Tu et al., 2018) (Dimitra et al., 2013) *DTI* + *DKI* (Allen et al., 2019)	–	*DTI* + *DKI* (Allen et al., 2019)
Attention and executive functions	*DTI (FA*↓*;MD,DA,DR↑)* (Gill et al., 2021) *FA, MD* + *DKI* (Allen et al., 2019)	–	–

**TABLE 8:** Correlation analysis between white matter parameters and affection scales in limbic fibers.

Affection			Fibers		
	**aCingulum**	**pCingulum**	**Fornix**	**UF**	**ATR**
Depression	*DTI (FA*↓*;MD,DA,DR↑)* (Duffy et al., 2014)	–	*DTI (FA*↓*;MD,DA,DR↑)* (Zhou et al., 2022a) (Duffy et al., 2014)	*DTI (FA*↓*;MD,DA,DR↑)* (Duffy et al., 2014)	*DTI (FA*↓*;MD,DA,DR↑)* (Zhou et al., 2022a)
Anxiety	*DTI (FA*↓*;MD,DA,DR↑)* (Tighe et al., 2012)	–	*DTI (FA*↓*;MD,DA,DR↑)* (Tighe et al., 2012)	–	–
Apathy	*DTI (FA*↓*;MD,DA,DR↑)* (Tighe et al., 2012)	–	*DTI (FA*↓*;MD,DA,DR↑)* (Tighe et al., 2012)	–	*Volume* (Torso et al., 2015)

**TABLE 9:** Correlation analysis between white matter parameters and affection scales in projection fibers.

Affection			Fibers			
	**ACR**	**SCR**	**PCR**	**PLIC**	**RIC**	**CP**
Depression	*Volume* (Fujishima et al., 2014)	*DTI (FA*↓*;MD,DA,DR↑)* (Duffy et al., 2014)	*DTI (FA*↓*;MD,DA,DR↑)* (Duffy et al., 2014) *Volume* (Fujishima et al., 2014)	–	–	–
Anxiety	–	–	–	–	–	*DTI (FA*↓*;MD,DA,DR↑)* (Tighe et al., 2012)
Apathy	–	–	–	–	–	*DTI (FA*↓*;MD,DA,DR↑)* (Tighe et al., 2012)

## Discussion

This review evaluated the relationship between white matter disconnections and behavioral disturbances in MCI. The white matter connections were classified into limbic, projection, association, and commissural fibers according to their connecting brain regions. The correlation studies on diffusion parameters of white matter and the behavior test scales were performed. Cognitions such as episodic memory, semantic memory, visuospatial, attention and executive functions were mainly related to the limbic, association, and commissural fibers. Affections such as depression, anxiety, and apathy are primarily associated with white matter disconnections in the limbic and projection fibers.

### Hippocampus and temporal lobe related fibers associated with cognition

Memory loss is the principal clinical manifestation of MCI (Gainotti et al., 2014). The hippocampus is responsible for memory as a critical limbic system component (Bender et al., 2020). Studies on molecular biomarkers, gray matter structure, and functional networks suggest that white matter fibers connected to the hippocampus and temporal lobe appear to be the earliest degeneration in MCI.

#### Molecular deposition evidence

Molecular biomarkers studies show that the medial temporal lobe and hippocampus are vital sites for amyloid β (Aβ) and hyperphosphorylated tau (pTau) deposition during MCI. Aβ and Tau are detected near the hippocampus before the MCI phase (Rieckmann et al., 2016; Rabin et al., 2019) and deposited in the temporal lobe region near the hippocampus during the MCI (Blamire, 2018). From MCI to AD, the deposition of Aβ and Tau spreads from the medial temporal lobe to the precuneus in the parietal lobe (Pegueroles et al., 2017; Rabin et al., 2019). Aβ and Tau deposition has been found to lead to the demyelination of white matter fibers (Jagust, 2018). Aβ and Tau are deposited first in the hippocampus and temporal lobe during MCI, damaging the white matter structures connected to the hippocampus and temporal lobe.

#### Gray matter atrophy evidence

Gray matter structure studies have revealed that structures in the temporal lobe, especially the hippocampus, are the critical area of gray matter atrophy during MCI (Jack et al., 2012; Brueggen et al., 2019). In MCI, gray matter atrophy begins with the hippocampus and gradually spreads to the entorhinal cortex, amygdala and other parahippocampal tissues in the temporal lobe (Lee et al., 2014; Lombardi et al., 2020). The hippocampus in the temporal lobe is connected to the parahippocampal tissues by white matter fibers (Zhuo et al., 2016). It was found that the damage to the white matter is an essential cause of the gray matter atrophy (Agosta et al., 2011). The white matter fibers connecting the hippocampus to the temporal lobe first degenerate during MCI and cause gray matter atrophy in the hippocampus and temporal lobe.

#### Functional connectivity declined evidence

Functional network studies have shown that the functional connectivity between the hippocampus and temporal lobe is significantly reduced in MCI (Lee et al., 2014). In the medial temporal lobe, the amygdala and parahippocampal gyrus have decreased functional connectivity with the hippocampus in MCI (Cai et al., 2017). The medial temporal lobe is an important component of the DMN network, closely related to memory (Li X. et al., 2020). In addition, the hippocampus has decreased functional connectivity with the superior and middle temporal gyrus in the temporal lobe (Liu et al., 2021). The medial and superior temporal gyrus are involved in cognitions such as language comprehension. It has been found that the degeneration of white matter fibers causes the weakening of functional connectivity (Vazquez-Rodriguez et al., 2019). The weakened functional connectivity between the hippocampus and temporal lobe in MCI suggests the white matter disconnections between the hippocampus and temporal lobe.

Therefore, white matter abnormalities related to the hippocampus and temporal lobe are associated with cognition decline in MCI.

### Thalamus related fibers–common pathways for cognition and affection

The main cause of cognition decline and affection abnormalities is the white matter disconnection of the neuronal pathways (Jiang and Lou, 2023). The thalamus serves as a relay station for transmitting information from subordinate neurons to the cerebral cortex. The thalamus has extensive white matter connections to the subcortical nuclei and the cerebral cortex (Abivardi and Bach, 2017). The studies on gray matter structure and functional network suggest abnormalities in the thalamus related fibers in MCI.

#### Gray matter atrophy evidence

Studies on gray matter have revealed that the ventral medial thalamic area undergoes atrophy during the MCI (Nie et al., 2017). Furthermore, gray matter atrophy progressed to the frontal and parietal lobes during AD (Gong et al., 2017). It has been confirmed that white matter degeneration precedes the atrophy of gray matter (Zhuang et al., 2012a; Jack and Holtzman, 2013). Additionally, the projection fibers connect the dorsolateral thalamic area with the parietal and frontal lobes, indicating a potential degeneration of the projection fibers that connect the subcortical nuclei to the cerebral cortex.

#### Functional connectivity declined evidence

Functional network studies have shown that functional connectivity between the thalamus and the medial temporal, prefrontal and precuneus brain regions in the default network is reduced in MCI (Cai et al., 2015). Reduced functional connectivity between the thalamus and the medial temporal lobe affects the memory capacity of MCI (Min et al., 2019). Reduced functional connectivity between the thalamus and the prefrontal and precuneus affects executive and emotion in MCI (Fjell et al., 2017; Scott et al., 2017). In AD, the functional connectivity between the thalamus and the frontal and parietal lobes is further reduced, leading to aphasia, dysfunction, and dyscognition (Raj et al., 2015). It has been shown that white matter degeneration causes functional connectivity to weaken (Vazquez-Rodriguez et al., 2019). The reduced functional connectivity suggests projection fibers connecting the thalamus to the frontal and parietal lobes may abnormal.

#### Comorbidity fiber pathways

Besides cognition decline, MCI often suffer from depression, anxiety, apathy and other affection symptoms (Velayudhan, 2023). The thalamus is responsible for memory processing in the limbic system together with the hippocampus. Meanwhile, the thalamus is connected to the amygdala, insula, anterior cingulate gyrus, and parts of the frontal lobe through the projection fibers, which are responsible for affection regulation. Damage to the white matter pathway of the projection fibers between the thalamus and the medial frontal lobe leads to a disruption of information transmission between the cortex and subcortical nuclei, altering the response to external stimuli and increasing the likelihood of cognition and affection abnormalities (Korgaonkar et al., 2014, Yatawara et al., 2019).

Therefore, the white matter degenerations of the thalamus related fibers are associated with cognition decline as well as affection abnormalities in MCI.

### Prediction for cognition and affection is crucial for AD early diagnosis

Although clinicians can currently screen MCI with behavior scales, relying on behavior scales alone to confirm MCI is too subjective. It is insensitive to detecting early symptoms of AD influenced by individual differences. Therefore, a more objective, accurate and reliable method is needed to identify and diagnose MCI in the early stage.

#### Artificial intelligence applied in MCI prediction

The ultimate goal of neuroimaging is to provide physicians with an objective diagnostic basis for screening, diagnosis, and prediction. Data-driven approaches have emerged as a new way of early diagnosis of MCI (Mechelli and Vieira, 2019). Research on individualized prediction based on neuroimaging is increasing yearly, with the prediction of cognition and affection accounting for the current research hotspot (Sui et al., 2020). For cognition and affection in MCI, artificial intelligence (AI) algorithms can perform in-depth analysis based on patients’ multidimensional data such as biomarkers, neuroimaging, and behavioral measures (Dwyer et al., 2018). In addition, AI algorithms can reduce the interference of subjective factors, optimize the model and improve the precision of prediction (Auffermann et al., 2019).

#### Feature extraction

The selection of the appropriate modality in the acquired imaging data and the accurate feature extraction method is usually more important than the underlying algorithm (Zhang et al., 2021; Zhou et al., 2022b). The methods for extracting white matter information in MCI brain images include reduced density map feature-based methods, predefined region-based methods, discriminative voxel selection-based methods, and connectivity network measure-based methods (Rathore et al., 2017). In addition, multimodal data provide a wider variety of features for MCI prediction. Previous studies have combined structural, functional, and diffusion MRI brain imaging features. Capturing disease information from different modalities and complementary features from multiple perspectives, thus enhancing model performance (Venugopalan et al., 2021).

#### Algorithms for prediction

Kernel functions and partial least squares correlation analysis capturing the relationship between white matter features and behavior is an effective measure to predict cognition and affection in MCI (Rashid and Calhoun, 2020). Kernel function-based methods often use a local linear weighted regression model that assigns weights to data points using a Gaussian kernel near each prediction point (Mihalik et al., 2020). Partial least squares methods often use regularization to reduce model overfitting by introducing penalty factors to constrain regression coefficients (Koutsouleris et al., 2018). In addition, using multimodal data to establish multivariate maps of different characteristics and behaviors can effectively improve the fit of regression models (Sui and Qi, 2018).

#### Generalization

The generalization of a model indicates the degree to which a statistical model generated in a set of data performs accurately in a new group or individual. The current scheme to support generalization is nested cross-validation (CV), where a training set is used internally to loop CV with the validation set to select the optimal parameters of the model. A test set is used externally to loop CV to obtain the model performance at the average level (Zhao et al., 2020). The CV has a generalization hierarchy with single-site CV, pooled multisite CV, leave-site-out CV, external validation and prospective validation in descending order of test stringency, with the most stringent being validation of unknown individuals (Dwyer et al., 2018). The training of generalized models relies on a multisite database of multiple samples. The Alzheimer’s disease neuroimaging initiative (ADNI) has a large publicly available dataset with brain imaging data from diagnosed AD, MCI, and healthy controls (Weber et al., 2021). Using a multicenter, extensive sample database with nested CV as a technical tool can fully ensure the model’s generalization (Dou et al., 2020).

Therefore, AI technology has a broad application prospect in the early diagnosis and treatment of MCI, which deserves further exploration and research.

### Limitations and perspectives

Exploring the relationship between white matter disconnections and behavioral disturbances such as cognition and affection in MCI is a hot topic of current research. However, there are currently the following problems:

Firstly, studies of specific fibers have focused only on changes in white matter parameters on single nerve tracts in MCI and lack comprehensiveness of the global degenerative mechanisms of MCI. Secondly, it leads to difficulties in feature extraction due to the lack of quantitative indicators for the global white matter network composed of specific fibers. Previous studies have focused on brain regions and network nodes. Quantitative descriptions of neuronal pathway disconnections were laked to analyze the intrinsic relationships between nodes and edges in the network.

Furthermore, most of the studies used small sample sizes for the datasets. There was a significant negative correlation between model prediction accuracy and sample size. Better predictions manifest likely on small samples, which indicate overfitting in the construction of the model. Finally, predicting MCI cognition and affection requires regression models between features and behavior. However, multimodal data with high-dimensional data have interdependent complex multivariate relationships. The optimization method to select relevant variables by constraints is computationally intensive, and the correlation between features is weak. There is a lack of a multivariate regression model based on a comorbid pathway of cognition and affection in MCI to establish the mapping relationship between features and behaviors effectively.

In the future, the sample set should be expanded to analyze changes in specific fibers using multimodal data. White matter networks should combine with graph theory analysis. The edge-centered network clustering approach should be used to extract the combined features of multiple fibers to predict behavioral disturbances such as cognition and affection in MCI.

## Conclusion

This article reviews the recent 12 years of studies using diffusion MRI techniques on white matter disconnections associated with behavioral impairment in MCI. The studies showed that degenerated fibers related to the hippocampus and temporal lobe were associated with cognition decline in MCI. Degenerated fibers related to the thalamus were associated with both cognition decline and affection abnormalities in MCI. The sensitivity of diffusion MRI to fiber microstructures can provide a reliable indicator of white matter disconnections in MCI, which can be further quantified in combination with behavioral scales of the patients. This review integrated the correspondence between specific fibers in MCI and behavioral disturbances, which provides a theoretical basis for the subsequent early diagnosis and targeted treatment of AD.

## Author contributions

YZ designed and conceptualized the research. YZ, LW, and SG acquisition, analysis, and interpretation of literature. YZ wrote the manuscript. JW and ZH supervision. All authors contributed to the article and approved the submitted version.
